# The associations between fresh vegetable and fruit consumption and plasma and PET biomarkers in preclinical Alzheimer's disease: A cross-sectional and longitudinal study of Chinese population

**DOI:** 10.1016/j.tjpad.2025.100076

**Published:** 2025-01-30

**Authors:** Heling Chu, Chuyi Huang, Fang Xie, Qihao Guo

**Affiliations:** a Department of Gerontology, Shanghai Sixth People's Hospital Affiliated to Shanghai Jiao Tong University School of Medicine, Shanghai, China; b Health Management Center, Renji Hospital, School of Medicine, Shanghai Jiaotong University, Shanghai, China; c PET Center, Huashan Hospital, Fudan University, Shanghai, China

**Keywords:** Alzheimer's disease, Vegetables and fruits, Older cognitively unimpaired adults, ^18^F-florbetapir PET

## Abstract

**Background:**

The identification of the modifiable lifestyle factors including dietary habits in older adults of preclinical Alzheimer's disease (AD) and early effective interventions are of great importance.

**Objectives:**

We studied whether the consumption of fresh vegetables and fruits was different between cognitively unimpaired (CU) and cognitively impaired (CI) population and mainly investigated the associations between vegetable and fruit consumption and PET and plasma AD biomarkers in older CU adults with higher β-amyloid (Aβ) burden.

**Design, setting, and participants:**

Older adults with the age of 50–85 years were enrolled for a cross-sectional and longitudinal study. The groups depended on whether the participants were CU or CI. Partial participants whose habits remained unchanged were followed up.

**Measurements:**

The consumption data of vegetables and fruits were collected using a validated self-reported questionnaire. We mainly investigated the associations between vegetable and fruit consumption and various biomarkers in CU participants with positive ^18^F-florbetapir PET scan (Aβ-PET), part of whom also underwent plasma AD biomarkers tests and ^18^F-MK6240 PET scan (tau-PET). Correlation and multiple linear regression analyses were used to investigate the associations between vegetable and fruit consumption and AD biomarkers.

**Results:**

A total of 1433 participants were enrolled, of which CU accounted for 49.4 %. Most of the intake habits of vegetables and fruits was different between CU and CI participants. 177 CU participants with Aβ-PET positive were selected for the following study. Multiple linear regression analysis showed higher consumption of fresh vegetables (>200 g/d), dark vegetables (>100 g/d, ≥2d/week), fruits (>100 g/d), berries (>100 g/d) and grapes (>100 g/d) more or less had associations with the plasma biomarkers including Aβ40, t-Tau, p-Tau-181 and neurofilament light chain as well as amyloid and Tau PET biomarkers. Most of the habits were associated with the change of cognitive function after an approximately two-year follow-up. Especially, higher intakes of fruits and grapes correlated with both lower Aβ and Tau burden and inversely with cognitive decline after follow-up.

**Conclusion:**

Our data indicates that higher consumption of vegetables, dark vegetables, fruits, berries and grapes is associated with amyloid and Tau PET and plasma biomarkers in preclinical AD participants and the changes of cognitive function after follow-up. Higher intakes of fruits (>100 g/d) and grapes (>100 g/d) may be more helpful for reducing the risk of AD development.

## 1 Introduction

Alzheimer's disease (AD) accounts for 60 % to 70 % of all dementia cases, which is the most prevalent cause of dementia [1]. The pathology of AD is charactered by the accumulation of amyloid-β (Aβ) peptides, sequestered into extracellular plaques, and intracellular neurofibrillary tangles comprising tau protein [2]. AD is a continuum with an extended pre-symptomatic phase although the accumulation of Aβ already starts, which is also called preclinical AD [3]. As the continuous Aβ accumulation accelerates cognitive decline, timely intervention to the risk factors that may faciliate the development of AD pathology in people who were cognitively unimpaired (CU) can contribute beneficial effects. [4] Evidences have supported that lifestyle modifications ameliorate the cognitive and functional trajectories across the AD spectrum [5]. As a result, the identification and interventions to the modifiable lifestyle-related risk factors in preclinical AD may contribute promising early prevention.

It has been revealed that special dietary habits are associated with less cognitive decline and a lower risk of AD [6]. Also, nutrient intervention may elicit beneficial effects on cognitive function. [7] It is demonstrated that higher intakes of vegetables and their constituent nutrients are associated with a lower risk of dementia in older adults [8]. Meanwhile, higher green leafy vegetable intake is indicated to be associated with less AD pathology in postmortem brain tissue of older adults [9]. Furthermore, increased consumption of fruits is associated with a reduced risk of cognitive impairment and dementia [10]. However, few studies have concerned the relationship between vegetable and fruit consumption and AD biomarkers in older CU participants with positive Aβ-positron emission tomography (PET).

As the 2024 revised criteria for diagnosis and staging of AD by Alzheimer's Association Workgroup, amyloid PET is regarded as a Core 1 biomarker that defines the initial stage of AD that is detectable *in vivo* and can identify the presence of AD in both symptomatic and asymptomatic individuals. Tau PET is a Core 2 biomarker which becomes abnormal later in the evolution of AD and is more closely linked with the onset of symptoms [11]. Although further validation is still required, blood-based biomarkers have their special advantages compared with cerebrospinal fluid (CSF) and PET biomarkers, such as minimally invasive, safe, less resource-intensive and broadly available. The main blood-based biomarkers tested in our work included plasma Aβ42/40, neurofilament light chain (NfL) and phosphorylated Tau-181 (p-Tau-181). These biomarkers on one hand are promising to uncover *in vivo* cerebral pathologies of AD as they are reported to be in agreement with the AT(N) categories moderately to excellently. It has been demonstrated that plasma Aβ42/40 is associated with Aβ pathology (A); plasma p-Tau correlates with Tau-mediated pathology (T); and plasma NfL levels correlate with axonal damage and neurodegeneration (N) [12]. On the other hand, the blood-based biomarkers have potential to indicate or prognosticate AD progression. Studies have revealed that low plasma Aβ42/40 is associated with more pronounced decline in cognitive function over time [13]. Meanwhile, NfL demonstrates predictive capabilities for decline of global cognition and multiple subdomains in the AD continuum [14]. Moreover, high plasma p-Tau-181 is associated with subsequent development of AD dementia in CU and MCI subjects [15]. The core focus of our study was the associations between vegetable and fruit consumption and AD pathology and disease progression. As a result, we also studied the change of plasma biomarkers in addition to the well-known PET biomarkers.

In the current study, we first studied whether the consumption of fresh vegetables and fruits evaluated by a self-reported questionnaire was different between CU and cognitively impaired (CI) population. We further divided the CU and CI participants undergoing Aβ-PET into subgroups depending on the positivity of Aβ-PET and compared the above habits. Then the associations between vegetable and fruit consumption and PET and plasma AD biomarkers in older CU adults with higher Aβ burden verified by positive of Aβ-PET were mainly investigated via a cross-sectional and longitudinal study. A part of the participants completed Tau-PET scan which is defined as a Core 2 biomarker [11]. The aim was to find the useful intake habits of vegetables and fruits which may be associated with less burden of AD pathology.

## 2 Methods

### 2.1 Study participants

Participants of Chinese Preclinical Alzheimer's Disease Study (C-PAS) cohort from Shanghai Sixth People's Hospital Affiliated to Shanghai Jiao Tong University School of Medicine between January 1, 2019 and December 31, 2023 were enrolled for analysis. The inclusion and exclusion criteria were according to our previous research [16]. Inclusion criteria: (1) aged 50–85 years; (2) education ≥1 year; (3) visual and auditory acuity adequate for testing; (4) completed the questionnaires for detailed data of fresh vegetable and fruit consumption. Exclusion criteria: (1) history of significant systemic or chronic illness; (2) history of major psychological disorders; (3) history of significant neurologic disease (such as stroke, brain trauma, subdural hematomas, hydrocephalus, epilepsy, intracranial tumors and infections); (4) other conditions that may affect cognitive function (such as alcoholism, drug abuse, abnormalities in folic acid, vitamin B12 and thyroid function); (5) refused to be enrolled. ^18^F-florbetapir PET scan was performed in part of the participants for detecting the deposition of Aβ. The participants were divided into two groups depending on whether they had completed Aβ-PET scan or not and the participants undergoing Aβ-PET, about a half, were divided into Aβ-PET positive and negative groups. Each group was further divided into CU and CI groups. CU was determined when (1) education-adjusted performance for the Chinese version of Mini-Mental State Examination (MMSE) (>24 for >6 years of education, >20 for 1–6 years of education) [17]; (2) failure to meet the diagnostic criteria of mild cognitive impairment (MCI) [18] or dementia [19]. CI contained MCI and dementia. Plasma AD biomarkers were tested by using single molecule array (Simoa) and ^18^F-MK6240 PET were performed for detecting Tau deposition in a proportion of CU participants with positive Aβ-PET. The follow-up study was conducted in part of preclinical AD individuals whose habits of vegetable and fruit consumption remained unchanged for determining the changes of cognitive function, plasma biomarkers and Aβ burden. The groups and study protocol were shown in eFig. 1. Full written informed consent was obtained from all participants or their next-of-kin prior to the first study procedure. Ethical approval was granted by the Ethics Committee of Shanghai Sixth People's Hospital Affiliated to Shanghai Jiao Tong University School of Medicine.

### 2.2 Clinical data and cognitive assessment

The essential clinical data containing age, sex, years of education, body mass index (BMI), the history of smoking, alcohol consumption, hypertension, diabetes mellitus, hyperlipidemia and coronary heart disease were recorded. Also, the participants were classified as either *apolipoprotein E* (*APOE*) ε4 carriers or noncarriers based on *APOE* genotype. General cognitive performance was assessed by MMSE and Chinese version of Montreal Cognitive Assessment-Basic (MoCA-BC) [20]. We also performed a battery of standardized neuropsychological tests including Auditory Verbal Learning Test (AVLT) 30-min delayed free recall and AVLT recognition[21], Boston Naming Test (BNT) [22], Animal Verbal Fluency Test (AFT) [23], Shape Trail Test Part A and B (STT-A and STT-B) [24] to determine whether any cognitive domain was impaired.

### 2.3 Fresh vegetable and fruit consumption

The consumption data of fresh vegetables and fruits were collected by trained research staff using a validated self-reported questionnaire according to the habits of Chinese population (eTable 1). The questionnaires were completed by the participants and their next of kin and/or caregivers who were very familiar with the participants’ dietary habits. We divided each item into two levels, of which moderate to high intake was defined as follows: vegetable consumption>200 g/d; dark vegetable consumption>100 g/d; fruit consumption>100 g/d; berry consumption>100 g/d; grape consumption>100 g/d. The questionnaire was completed according to the participant's dietary habits within the past 3 months [25,26].

### 2.4 Amyloid and Tau PET imaging

In order to evaluate the brain Aβ and Tau burden, the ^18^F-florbetapir and ^18^F-MK6240 PET scans were performed by using a PET/CT system (Biograph mCT Flow PET/CT, Siemens, Erlangen, Germany) at the PET center of Huashan hospital, Fudan University. Cerebral amyloid PET scans were carried out 50 min after the intravenous injection of 7.4 MBq/kg (0.2 mCi/kg) florbetapir and lasted for 20 min. PET images were reconstructed by means of filtered back projection (FBP) algorithm with corrections for decay, normalization, dead time, photon attenuation, scatter and random coincidences. PET images were then coregistered to the individual structural MRI and spatially normalized in the Montreal Neurological Institute (MNI) template. Standard uptake value ratios (SUVRs) in the cortical regions of interest (ROIs) consisting of frontal gyrus, lateral parietal gyrus, lateral temporal gyrus, medial temporal gyrus, posterior cingulate gyrus, and precuneus were calculated by using cerebellar crus as a reference. Global SUVR values were calculated by weighted averaging of these ROIs [27]. The positive ^18^F-florbetapir PET images were defined by the method of visual rating according to the guidelines for interpreting amyloid PET [28]. Tau PET imaging involved the intravenous bolus injection of 5.55 MBq/kg ^18^F-MK6240 with a 20-min acquisition time commencing 90 min post injection. Attenuation correction was performed with low dose CT projection scanning. Images were reconstructed by FBP and pretreated by statistical parametric mapping 12 software. The inferior cerebellum was used as the reference region for calculating ^18^FMK-6240 SUVR. Tau positivity was defined as 2.5 standard deviations (SDs) higher than the mean SUVR of young CU individuals for each ROI [29]. PET Braak-like stages were defined as follows: Braak I (transentorhinal), Braak II (entorhinal and hippocampus), Braak III (amygdala, parahippocampal gyrus, fusiform gyrus and lingual gyrus), Braak IV (insula, inferior temporal, lateral temporal, posterior cingulate and inferior parietal), Braak V (orbitofrontal, superior temporal, inferior frontal, cuneus, anterior cingulate, supramarginal gyrus, lateral occipital, precuneus, superior parietal, superior frontal and rostro medial frontal) and Braak VI (paracentral, postcentral, precentral and pericalcarine) [30]. Braak-like stages were determined only if the current and previous stages were achieved while the later stages were negative [29]. The PET image interpretation was independently performed by three nuclear medicine specialists and the results depended on the agreement of more than two specialists.

### 2.5 Measurements of plasma AD biomarkers

Plasma AD biomarkers were tested by using Simoa according to our previous study [31,32]. In brief, whole blood samples were centrifugated at 500 × *g* for 5 min at 4 °C and subsequently aliquoted into ultra-low adsorption tubes (AXYGEN MCT-150-l-C) on ice and stored at −80 °C refrigerator. Plasma samples were transferred from the refrigerator to ice plate for 30 min, and then centrifuged at 10,000 × *g* for 5 min at 4 °C prior to testing. The measurements of plasma AD biomarkers contained Aβ42, Aβ40, t-Tau (Neurology 3-Plex A Assay Kit, Lot 502,838), p-Tau-181 (Assay Kit V2, Lot 502,923) and NfL (NF-light Assay Kit, Lot 202,700), which were performed on the Quanterix Simoa HD-1 platform [33]. Reagent pretreatment and sample loading were carried out according to the instructions of the manufacture. Concentrations of each biomarker (pg/mL) were determined by the four-parameter curve fit algorithm using Simoa HD-1 Analyzer software.

### 2.6 Statistical analysis

Statistical analyses were performed by using SPSS 22.0. Data for continuous variables were presented as means with standard deviations (SDs) or medians with interquartile ranges (IQRs) when appropriate and analyzed using two-tailed Student *t-*test, one-way analysis of variance (ANOVA), Mann-Whitney *U* test, or Kruskal-Wallis *H* test, which were determined by data distribution and the number of groups. Data for categorical variables were expressed as a percentage and compared using Chi-square test or Fisher exact test (2-tailed). Bonferroni corrections were used for multiple comparisons. Spearman's correlation analysis was used to correlate the dietary habits with each biomarker. The variables with significant correlation (*P* < 0.05) were then further analyzed by multiple linear regression. In the CU participants with Aβ-PET (+), we examined the associations between the vegetable and fruit consumption and various AD biomarkers by using a multiple linear regression model, which was adjusted for age, sex, education, BMI, *APOE* ε4 positive, MoCA-BC, smoking, alcohol consumption and the history of hypertension, diabetes mellitus, hyperlipidemia and coronary heart disease. The biomarkers included the blood-based, amyloid and Tau PET biomarkers as well as the changes of cognitive function and plasma and Aβ-PET biomarkers in the follow-up cohort (differences between the follow-up and baseline data). We reported unstandardized coefficient estimates (β) and standardized estimates (SE) with 95 % confidence intervals (CI) and significance test results (*P* values). Further subgroup analyses stratified by *APOE* ε4 genotype (*ε4* carriers, non-carriers) were conducted to explore the potential associations in the subgroups. The level of statistical significance was set at *P* < 0.05. All analyses were conducted by statisticians blinded to the groups.

## 3 Results

### 3.1 Comparison of the differences of vegetable and fruit consumption between CU and CI participants in each group

A total of 1433 participants (708 CU, 725 CI) were included in our study. CU participants exhibited higher vegetable, fruit, berry and grape consumption than CI participants. Moreover, higher proportion of vegetable consumption>200 g/d, dark vegetable consumption≥ 2 d/week, fruit consumption>100 g/d, berry consumption>100 g/d and grape consumption>100 g/d was also observed in CU than CI participants (Table 1). Aβ-PET was performed in 698 participants, 345 of which showed positive results (177 CU, 168 CI) and negative results was concluded in 353 participants (206 CU, 147 CI). 325 CU and 410 CI participants who did not undergo Aβ-PET scan were also enrolled. The differences of vegetable and fruit consumption between CU and CI participants were similar to the total participants especially in those with positive Aβ-PET (eTable 2).

**Table 1:** Comparison of the demographic and clinical characteristics between all the cognitively unimpaired and cognitively impaired participants.

Characteristics	CU (*n* = 708)	CI (*n* = 725)	*P*
Age, y, mean (SD)	64.8 (8.4)	68.8 (7.3)	<0.001
Sex, male, n (%)	236 (33.3)	257 (35.4)	0.389
Education, y, median (IQR)	12 (10–15)	10 (9–12)	<0.001
BMI, kg/m^2^, mean (SD)	23.4 (3.2)	23.5 (3.2)	0.635
Smoking, n (%)	83 (11.7)	100 (13.8)	0.258
Alcohol consumption, n (%)	116 (16.4)	121 (16.7)	0.615
Hypertension, n (%)	244 (34.5)	260 (35.9)	0.579
Diabetes mellitus, n (%)	78 (11.0)	89 (12.3)	0.458
Hyperlipidemia, n (%)	119 (16.8)	127 (17.5)	0.722
Coronary heart disease, n (%)	52 (7.3)	58 (8.0)	0.641
MMSE, median (IQR)	28 (27–29)	24 (19–27)	<0.001
MoCA-BC, median (IQR)	26 (24–27)	18 (13–22)	<0.001
Vegetable and fruit consumption			
Vegetable consumption, median (IQR)	2 (2–3)	3 (2–3)	<0.001
Vegetable consumption>200 g/d, n (%)	409 (57.8)	356 (49.1)	0.001
Frequency of dark vegetable consumption, median (IQR)	1 (1–2)	1 (1–2)	0.217
Dark vegetable consumption≥ 2 d/week, n (%)	647 (91.4)	627 (86.5)	0.004
Dark vegetable consumption, median (IQR)	1 (1–2)	2 (1–2)	0.442
Dark vegetable consumption>100 g/d, n (%)	566 (79.9)	561 (77.4)	0.256
Fruit consumption, median (IQR)	2 (2–3)	2 (2–3)	<0.001
Fruit consumption>100 g/d, n (%)	493 (69.6)	426 (58.8)	<0.001
Berry consumption, median (IQR)	2 (2–3)	3 (2–3)	<0.001
Berry consumption>100 g/d, n (%)	377 (53.2)	269 (37.1)	<0.001
Grape consumption, median (IQR)	3 (2–3)	3 (2–3)	<0.001
Grape consumption>100 g/d, n (%)	324 (45.8)	247 (34.1)	<0.001

BMI indicates body mass index; CU, cognitively unimpaired; CI, cognitively impaired; IQR, interquartile range; MMSE, Mini-Mental State Examination; MoCA-BC, Chinese version of Montreal Cognitive Assessment-Basic; SD, standard deviation.

### 3.2 Associations between the vegetable and fruit consumption and plasma biomarkers in participants with Aβ-PET (+)

Aβ-PET positive was detected in 177 CU participants with the average age of 65.3 years. 53 participants also completed Tau-PET scans. The detailed demographic and clinical characteristics of CU participants with Aβ-PET (+) were shown in Table 2.

**Table 2:** Demographic and clinical characteristics of the cognitively unimpaired participants with Aβ-PET (+).

Characteristics	
Baseline	
Clinical characteristics (*n* = 177)	
Age, y, mean (SD)	65.3 (7.1)
Sex, male, n (%)	78 (44.1)
Education, y, median (IQR)	12 (10–15)
BMI, kg/m^2^, mean (SD)	23.0 (2.7)
Smoking, n (%)	27 (15.3)
Alcohol consumption, n (%)	33 (18.6)
*APOE* ε4 carrier, n (%)	49 (27.7)
Hypertension, n (%)	56 (31.6)
Diabetes mellitus, n (%)	22 (12.4)
Hyperlipidemia, n (%)	21 (11.9)
Coronary heart disease, n (%)	13 (7.3)
Neuropsychological tests (*n* = 177)	
MMSE, median (IQR)	28 (27–29)
MoCA-BC, median (IQR)	25 (23–27)
AVLT delayed recall, median (IQR)	5 (3–7)
AVLT recognition, median (IQR)	22 (20–23)
BNT, median (IQR)	24 (22–26)
AFT, median (IQR)	16 (14–20)
STT-A, median (IQR)	45 (37–55)
STT-B, median (IQR)	121 (97–146)
Vegetable and fruit consumption (*n* = 177)	
Vegetable consumption (n, %)	
>300 g/d	42 (23.7)
200–300 g/d	63 (35.6)
100–200 g/d	57 (32.2)
<100 g/d	15 (8.5)
Frequency of dark vegetable consumption (n, %)	
≥4 d/week	119 (67.2)
2–3 d/week	48 (27.1)
1 d/week	8 (4.5)
<1 d/week	2 (1.2)
Dark vegetable consumption (n, %)	
>150 g/d	94 (53.1)
100–150 g/d	56 (31.6)
50–100 g/d	20 (11.3)
<50 g/d	7 (4.0)
Fruit consumption (n, %)	
>200 g/d	38 (21.5)
100–200 g/d	82 (46.3)
<100 g/d	43 (24.3)
No	14 (7.9)
Berry consumption (n, %)	
>200 g/d	29 (16.4)
100–200 g/d	59 (33.3)
<100 g/d	89 (50.3)
Grape consumption (n, %)	
>200 g/d	25 (14.1)
100–200 g/d	56 (31.6)
<100 g/d	96 (54.3)
Plasma biomarkers (*n* = 138)	
Aβ42 (pg/ml), mean (SD)	9.37 (3.74)
Aβ40 (pg/ml), mean (SD)	193.68 (69.35)
Aβ42/40, mean (SD)	0.050 (0.014)
t-Tau (pg/ml), median (IQR)	2.21 (1.68–2.95)
p-Tau-181 (pg/ml), median (IQR)	1.84 (1.25–2.61)
NfL (pg/ml), median (IQR)	14.70 (10.67–19.90)
PET biomarkers	
Aβ-PET SUVR (*n* = 177)	1.13 (1.06–1.27)
Tau-PET Braak stages (*n* = 53)	0 (0–2)
Follow-up	
Neuropsychological tests (*n* = 59)	
dMMSE, median (IQR)	0 (−2–1)
dMoCA-BC, median (IQR)	1 (−2–1)
Cognitive impairment (n, %)	11 (18.6)
Follow-up time (months), mean (SD)	28.9 (7.8)
Plasma biomarkers (*n* = 14)	
dAβ42 (pg/ml), mean (SD)	0.96 (5.23)
dAβ40 (pg/ml), mean (SD)	25.29 (115.75)
dAβ42/40, mean (SD)	−0.004 (0.009)
dt-Tau (pg/ml), median (IQR)	−0.31 (−0.61- −0.16)
dp-Tau-181 (pg/ml), median (IQR)	0.15 (−0.23–0.47)
dNfL (pg/ml), median (IQR)	−0.01 (−0.84–1.77)
Follow-up time (months), mean (SD)	23.7 (4.6)
Aβ-PET dSUVR (*n* = 24)	−0.007 (−0.614- −0.162)
Follow-up time (months), mean (SD)	28.5 (8.0)

Aβ indicates amyloid-β; AFT, Animal Verbal Fluency Test; APOE, apolipoprotein E; AVLT, Auditory Verbal Learning Test; BMI, body mass index; BNT, Boston Naming Test; IQR, interquartile range; MMSE, Mini-Mental State Examination; MoCA-BC, Chinese version of Montreal Cognitive Assessment-Basic; NfL, neurofilament light chain; PET, positron emission tomography; SD, standard deviation; STT, Shape Trail Test.

Plasma AD biomarkers were obtained from 138 individuals. Spearman's correlation was used to correlate the consumption of vegetables and fruits with each AD biomarker. The significant correlations included vegetable consumption and NfL (*P* = 0.015); vegetable consumption>200 g/d and NfL (*P* = 0.045); dark vegetable consumption≥ 2 d/week and p-Tau-181 (*P* = 0.016); dark vegetable consumption and Aβ42 (*P* = 0.025), Aβ40 (*P* = 0.046), p-Tau-181 (*P* = 0.016); dark vegetable consumption>100 g/d and p-Tau-181 (*P* = 0.003); fruit consumption and t-Tau (*P* = 0.030), p-Tau-181 (*P* = 0.021), NfL (*P* < 0.001); fruit consumption>100 g/d and NfL (*P* = 0.006); berry consumption and p-Tau-181 (*P* = 0.009), NfL (*P* < 0.001); berry consumption>100 g/d and Aβ40 (*P* = 0.046), NfL (*P* = 0.001); grape consumption and p-Tau-181 (*P* = 0.002), NfL (*P* = 0.002); grape consumption>100 g/d and p-Tau-181 (*P* = 0.010), NfL (*P* = 0.014). Multiple linear regression analyses revealed the independent associations between the vegetable and fruit consumption and plasma biomarkers were as follows: dark vegetable consumption and Aβ40 (*P* = 0.005), p-Tau-181 (*P* = 0.005); dark vegetable consumption>100 g/d and p-Tau-181 (*P* = 0.008); fruit consumption and t-Tau (*P* = 0.031), NfL (*P* = 0.024); berry consumption and p-Tau-181 (*P* = 0.002); grape consumption and p-Tau-181 (*P* = 0.006); grape consumption>100 g/d and p-Tau-181 (*P* = 0.006) (eTable 3).

In the participants who were *APOE* ε4 carriers, the only association was dark vegetable consumption and Aβ42/40 (*P* < 0.001) (eTable 4).

### 3.3 Associations between the vegetable and fruit consumption and amyloid and Tau PET biomarkers in participants with Aβ-PET (+)

Spearman's correlation followed by multiple linear regression analysis demonstrated that Aβ-PET SUVR was associated with vegetable consumption (*P* = 0.019), vegetable consumption>200 g/d (*P* = 0.012), frequency of dark vegetable consumption (*P* = 0.024), dark vegetable consumption (*P* < 0.001), fruit consumption (*P* = 0.013), berry consumption (*P* = 0.007), berry consumption>100 g/d (*P* < 0.001), grape consumption (*P* < 0.001), and grape consumption>100 g/d (*P* < 0.001). Besides, Tau PET Braak stages correlated with fruit consumption (*P* = 0.009), fruit consumption>100 g/d (*P* = 0.009), grape consumption (*P* = 0.015), and grape consumption>100 g/d (*P* = 0.032) (Table 3).

**Table 3:** Spearman's correlation and multiple linear regression analyzes of the association between vegetable and fruit consumption and Aβ-PET SUVR and Tau-PET Braak stages in cognitively unimpaired participants with Aβ-PET (+).

Variables	Biomarkers	Spearman's correlation	Multiple linear regression			
		Correlation coefficient (*P* value)	β	95 % CI	SE	*P* value
Vegetable consumption	Aβ-PET SUVR	−0.306 (<0.001)	−0.049	−0.089 to −0.008	0.020	0.019
	Tau-PET Braak stages	−0.198 (0.155)	/	/	/	/
Vegetable consumption>200 g/d	Aβ-PET SUVR	−0.313 (<0.001)	−0.096	−0.171 to −0.021	0.038	0.012
	Tau-PET Braak stages	−0.207 (0.136)	/	/	/	/
Frequency of dark vegetable consumption	Aβ-PET SUVR	−0.202 (0.011)	−0.063	−0.018 to −0.009	0.028	0.024
	Tau-PET Braak stages	−0.322 (0.019)	/	/	/	/
Dark vegetable consumption≥ 2 d/week	Aβ-PET SUVR	−0.145 (0.068)	/	/	/	/
	Tau-PET Braak stages	−0.090 (0.521)	/	/	/	/
Dark vegetable consumption	Aβ-PET SUVR	−0.186 (0.019)	−0.080	−0.123 to −0.038	0.022	<0.001
	Tau-PET Braak stages	−0.288 (0.036)	/	/	/	/
Dark vegetable consumption>100 g/d	Aβ-PET SUVR	−0.130 (0.102)	/	/	/	/
	Tau-PET Braak stages	−0.074 (0.601)	/	/	/	/
Fruit consumption	Aβ-PET SUVR	−0.266 (0.001)	−0.052	−0.092 to −0.011	0.021	0.013
	Tau-PET Braak stages	−0.342 (0.012)	−0.561	−0.969 to −0.152	0.200	0.009
Fruit consumption>100 g/d	Aβ-PET SUVR	−0.160 (0.044)	/	/	/	/
	Tau-PET Braak stages	−0.290 (0.035)	−1.022	−1.776 to −0.279	0.364	0.009
Berry consumption	Aβ-PET SUVR	−0.438 (<0.001)	−0.109	−0.154 to −0.064	0.023	0.007
	Tau-PET Braak stages	−0.284 (0.039)	/	/	/	/
Berry consumption>100 g/d	Aβ-PET SUVR	−0.397 (<0.001)	−0.162	−0.232 to −0.093	0.035	<0.001
	Tau-PET Braak stages	−0.281 (0.041)	/	/	/	/
Grape consumption	Aβ-PET SUVR	−0.356 (<0.001)	−0.093	−0.141 to −0.045	0.024	<0.001
	Tau-PET Braak stages	−0.438 (0.001)	−0.621	−1.111 to −0.131	0.240	0.015
Grape consumption>100 g/d	Aβ-PET SUVR	−0.314 (<0.001)	−0.132	−0.203 to −0.061	0.036	<0.001
	Tau-PET Braak stages	−0.446 (0.001)	−0.856	−1.633 to −0.078	0.381	0.032

Aβ indicates amyloid-β; CI, confidence intervals; PET, positron emission tomography; SE, standardized estimate; SUVR, standard uptake value ratio.

In the *APOE* ε4 carriers, higher fruit consumption was associated with lower Tau PET Braak stages while higher berry consumption was associated with lower Aβ-PET SUVR. Importantly, higher grape consumption correlated with both lower Aβ and Tau burden (eTable 4).

### 3.4 Associations between the vegetable and fruit consumption and the changes of cognitive function and biomarkers in participants with Aβ-PET (+)

A total of 59 participants whose habits of vegetable and fruit consumption were unchanged were followed up for investigating the associations between the vegetable and fruit consumption and the changes of cognitive function with the mean follow-up time of 28.9 months. The change of MoCA-BC was associated with frequency of dark vegetable consumption (*P* = 0.012), dark vegetable consumption≥ 2 d/week (*P* = 0.030), dark vegetable consumption (*P* = 0.005), fruit consumption (*P* = 0.002), fruit consumption>100 g/d (*P* = 0.005), berry consumption (*P* = 0.001), berry consumption>100 g/d (*P* = 0.001), grape consumption (*P* = 0.010), and grape consumption>100 g/d (*P* = 0.024). 11 CU participants (18.6 %) converted to CI patients and the conversion to CI was associated with dark vegetable consumption (*P* = 0.010). As to the plasma biomarkers, only the association between fruit consumption and the change of NfL was demonstrated (*P* = 0.021). In 24 participants with the mean follow-up time of 28.5 months, the change of Aβ-PET SUVR was associated with vegetable consumption (*P* = 0.001), vegetable consumption>200 g/d (*P* < 0.001), and berry consumption>100 (*P* = 0.032) (Table 4).

**Table 4:** Spearman's correlation and multiple linear regression analyzes of the associations between vegetable and fruit consumption and the changes of cognitive function and biomarkers in the longitudinal study of the cognitively unimpaired participants with Aβ-PET (+).

Variables	Biomarkers	Spearman's correlation	Multiple linear regression			
		Correlation coefficient (*P* value)	β	95 % CI	SE	*P* value
Vegetable consumption	dMMSE	0.144 (0.280)	/	/	/	/
	dMoCA-BC	0.127 (0.343)	/	/	/	/
	CI%	−0.101 (0.447)	/	/	/	/
	dAβ42/40	0.189 (0.536)	/	/	/	/
	dp-Tau-181	0.127 (0.665)	/	/	/	/
	dNfL	−0.396 (0.161)	/	/	/	/
	dSUVR	−0.534 (0.007)	−0.106	−0.162 to −0.050	0.026	0.001
Vegetable consumption>200 g/d	dMMSE	0.135 (0.312)	/	/	/	/
	dMoCA-BC	0.080 (0.548)	/	/	/	/
	CI%	−0.100 (0.453)	/	/	/	/
	dAβ42/40	0.178 (0.560)	/	/	/	/
	dp-Tau-181	0.166 (0.570)	/	/	/	/
	dNfL	−0.277 (0.337)	/	/	/	/
	dSUVR	−0.645 (0.001)	−0.287	−0.412 to −0.162	0.059	<0.001
Frequency of dark vegetable consumption	dMMSE	0.088 (0.514)	/	/	/	/
	dMoCA-BC	0.332 (0.011)	1.674	0.359 to 2.953	0.632	0.012
	CI%	−0.045 (0.733)	/	/	/	/
	dAβ42/40	−0.148 (0.629)	/	/	/	/
	dp-Tau-181	0.065 (0.825)	/	/	/	/
	dNfL	−0.228 (0.434)	/	/	/	/
	dSUVR	−0.482 (0.017)	/	/	/	/
Dark vegetable consumption≥ 2 d/week	dMMSE	0.196 (0.141)	/	/	/	/
	dMoCA-BC	0.292 (0.026)	3.507	0.354 to 6.660	1.556	0.030
	CI%	−0.167 (0.207)	/	/	/	/
	dAβ42/40	0.154 (0.615)	/	/	/	/
	dp-Tau-181	0.447 (0.109)	/	/	/	/
	dNfL	−0.447 (0.109)	/	/	/	/
	dSUVR	−0.392 (0.058)	/	/	/	/
Dark vegetable consumption	dMMSE	0.188 (0.157)	/	/	/	/
	dMoCA-BC	0.314 (0.016)	1.480	0.482 to 2.478	0.493	0.005
	CI%	−0.306 (0.018)	−0.158	−0.277 to 0.040	0.058	0.010
	dAβ42/40	−0.102 (0.740)	/	/	/	/
	dp-Tau-181	0.067 (0.820)	/	/	/	/
	dNfL	−0.132 (0.653)	/	/	/	/
	dSUVR	−0.388 (0.061)	/	/	/	/
Dark vegetable consumption>100 g/d	dMMSE	0.023 (0.863)	/	/	/	/
	dMoCA-BC	0.224 (0.092)	/	/	/	/
	CI%	−0.160 (0.226)	/	/	/	/
	dAβ42/40	−0.146 (0.633)	/	/	/	/
	dp-Tau-181	0.039 (0.894)	/	/	/	/
	dNfL	0.118 (0.689)	/	/	/	/
	dSUVR	−0.323 (0.124)	/	/	/	/
Fruit consumption	dMMSE	0.166 (0.214)	/	/	/	/
	dMoCA-BC	0.274 (0.037)	1.681	0.641 to 2.722	0.514	0.002
	CI%	−0.289 (0.026)	/	/	/	/
	dAβ42/40	0.061 (0.844)	/	/	/	/
	dp-Tau-181	−0.604 (0.022)	/	/	/	/
	dNfL	−0.621 (0.018)	−2.091	−3.767 to 0.414	0.727	0.021
	dSUVR	−0.500 (0.013)	/	/	/	/
Fruit consumption>100 g/d	dMMSE	0.133 (0.316)	/	/	/	/
	dMoCA-BC	0.360 (0.005)	2.682	0.843 to 4.520	0.907	0.005
	CI%	−0.344 (0.008)	/	/	/	/
	dAβ42/40	0.098 (0.751)	/	/	/	/
	dp-Tau-181	−0.706 (0.005)	/	/	/	/
	dNfL	−0.588 (0.027)	/	/	/	/
	dSUVR	−0.511 (0.011)	/	/	/	/
Berry consumption	dMMSE	0.099 (0.455)	/	/	/	/
	dMoCA-BC	0.354 (0.006)	2.237	0.997 to 3.477	0.612	0.001
	CI%	−0.278 (0.033)	/	/	/	/
	dAβ42/40	0.102 (0.741)	/	/	/	/
	dp-Tau-181	−0.231 (0.426)	/	/	/	/
	dNfL	0.027 (0.928)	/	/	/	/
	dSUVR	−0.615 (0.001)	/	/	/	/
Berry consumption>100 g/d	dMMSE	0.119 (0.368)	/	/	/	/
	dMoCA-BC	0.328 (0.011)	3.421	1.526 to 5.316	0.936	0.001
	CI%	−0.313 (0.016)	/	/	/	/
	dAβ42/40	−0.082 (0.789)	/	/	/	/
	dp-Tau-181	−0.159 (0.586)	/	/	/	/
	dNfL	0.053 (0.857)	/	/	/	/
	dSUVR	−0.758 (<0.001)	−0.162	−0.308 to −0.016	0.069	0.032
Grape consumption	dMMSE	0.131 (0.323)	/	/	/	/
	dMoCA-BC	0.356 (0.006)	1.681	0.429 to 2.932	0.619	0.010
	CI%	−0.145 (0.273)	/	/	/	/
	dAβ42/40	0.130 (0.673)	/	/	/	/
	dp-Tau-181	−0.280 (0.332)	/	/	/	/
	dNfL	−0.220 (0.449)	/	/	/	/
	dSUVR	−0.291 (0.167)	/	/	/	/
Grape consumption>100 g/d	dMMSE	0.115 (0.385)	/	/	/	/
	dMoCA-BC	0.286 (0.028)	2.330	0.321 to 4.340	0.993	0.024
	CI%	−0.139 (0.295)	/	/	/	/
	dAβ42/40	0.041 (0.894)	/	/	/	/
	dp-Tau-181	−0.251 (0.387)	/	/	/	/
	dNfL	−0.251 (0.387)	/	/	/	/
	dSUVR	0.378 (0.068)	/	/	/	/

Aβ indicates amyloid-β; CI, confidence intervals; CI%, conversion rate of cognitive impairment; “d”, difference between the follow-up and baseline data; MMSE, Mini-Mental State Examination; MoCA-BC, Chinese version of Montreal Cognitive Assessment-Basic; NfL, neurofilament light chain; PET, positron emission tomography; SE, standardized estimate; SUVR, standard uptake value ratio.

Only berry consumption and berry consumption>100 g/d were associated with cognitive decline in the *APOE* ε4 carriers (eTable 4).

### 3.5 The beneficial intake habits of vegetables and fruits in the current study

**Fig. 1:**
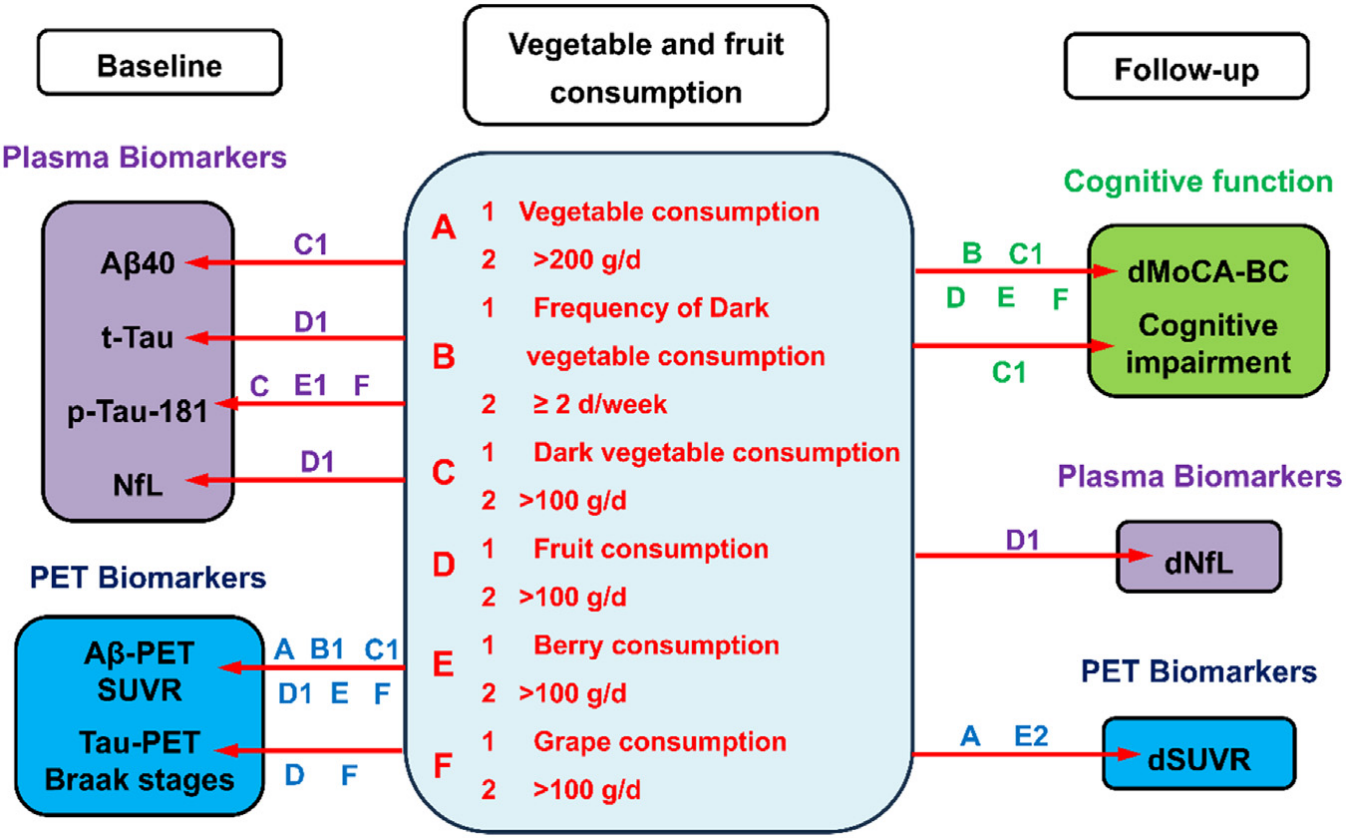
The associations between vegetable and fruit consumption and the biomarkers in the cross-sectional study as well as the changes of cognitive function and biomarkers in the longitudinal study of the cognitively unimpaired participants with Aβ-PET (+). If the associations were simultaneously observed in both “1″ and “2″ of each section, only the capital letters (A-F) are labeled. “d”, difference between the follow-up and baseline data; MoCA-BC, Chinese version of Montreal Cognitive Assessment-Basic; NfL, neurofilament light chain; PET, positron emission tomography; SUVR, standard uptake value ratio.

Fig. 1 summarizes the beneficial intake habits of vegetables and fruits and the corresponding biomarkers in participants with Aβ-PET (+) according to the findings of current work. Briefly, higher consumption of vegetables (>200 g/d), dark vegetables (>100 g/d), fruits (>100 g/d), berries (>100 g/d) and grapes (>100 g/d) as well as higher frequency of dark vegetable consumption (≥2 d/week) were associated with the PET and plasma biomarkers. Moreover, part of these habits also had associations with better cognitive function and lower Aβ burden after a two-year follow-up.

## 4 Discussion

Our current study including 1433 participants of Chinese population demonstrated that CU participants consumed higher vegetables, fruits, berries and grapes than CI participants in the total participants and each subgroup. Subsequently, we investigated the associations between vegetable and fruit consumption and plasma and PET biomarkers in 177 older CU adults with higher Aβ burden. The results indicated that higher consumption of vegetables (>200 g/d), dark vegetables (>100 g/d), fruits (>100 g/d), berries (>100 g/d) and grapes (>100 g/d) as well as higher frequency of dark vegetable consumption (≥2 d/week) more or less had associations with the plasma and amyloid and Tau biomarkers. After a two-year follow-up, part of the above habits were also associated with better cognitive function and lower Aβ burden. These findings have not been reported previously, which suggests that the above intake habits of vegetables and fruits could be considered as protective factors for AD pathology.

A large amount of evidence demonstrates that dietary intervention like Mediterranean diet (rich in fruits and vegetables) is associated with a slower rate of cognitive decline [34]. A study including 581 autopsied participants shows that higher green leafy vegetable intake is associated with less AD pathology among multiple dietary components [9]. Moreover, a 4-year randomized clinical trial (RCT) with 1401 participants demonstrates aerobic exercise and healthy diet (including ≥400 g/d of vegetables, fruit, and berries) interventions improve cognition in older individuals [35]. Berries, encompassing strawberry, blackberry, cranberry, blueberry, grape, etc., are a valuable source of bioactive metabolites that may have therapeutic potential in age-related neurological disorders including AD [36]. Grapes are the most popular berry consumed by Chinese population. As a result, we selected the above dietary components in the questionnaire. Especially, there is a wealth of evidence supporting the association between grape and cognitive function. A systematic review of RCTs suggests that grapes might have a positive effect on cognitive health after both acute and chronic interventions [37]. Grapes are a rich source of bioactive compounds, including anthocyanins, flavonoids, catechins, procyanidins, organic acids, and stilbenes, thus besides fresh grapes, their derivatives also contribute to cognitive improvement [38]. It has been demonstrated that Concord grape juice can enhance neurocognitive function in older adults with mild memory decline [39]. Other intervention of RCT include that a commercial grape extract supplementation (Cognigrape®) has both short- and long-term improvement of cognitive function in a healthy aging population [40]. The above findings are in agreement with our results. We mainly studied the associations between the consumption of vegetables and fruits and AD by using multiple reliable biomarkers, which few studies have involved. The results provide more evidence in the mechanisms of these habits to prevent AD.

We further conducted subgroup analysis stratified by *APOE* ε4 genotype. *APOE* ε4 is the most important genetic risk factor for AD and the carriers have a higher lifetime incidence of AD [41]. For *APOE* ε4 carriers, beneficial intake habits of vegetables and fruits were different from total participants. Previous study has also indicated that healthy dietary pattern plays different roles in reducing risk of dementia between *APOE* ε4 carriers and non-carriers [42]. The *APOE* ε4 allele is associated with hypercholesterolemia, insulin resistance, chronic inflammation and atherosclerosis [43], which are also risk factors of AD, thus *APOE* ε4 carriership may diminish the effect of vegetable and fruit consumption on reducing dementia risk. In our study, higher fruit, berry and grape consumption was associated with lower Aβ and/or Tau burden and higher berry consumption conversely correlated with cognitive decline after follow-up in *APOE* ε4 carriers, suggesting that these dietary habits may be more beneficial for reducing the risk of AD.

According to the 2024 revised criteria for diagnosis and staging of AD by Alzheimer's Association Workgroup, Core 1 biomarkers including Aβ-PET reflect the presence of AD neuropathologic change and is sufficient to establish a diagnosis of AD [11]. The participants in the comprehensive study were all Aβ-PET positive, indicating we investigated the associations in a biological AD population. Moreover, we also performed Tau-PET in part of the participants. Tau-PET is a Core 2 biomarker that provides prognostic information and may increase confidence that AD is contributing to symptoms [11]. The findings revealed that higher fruit and grape consumption was associated with lower Aβ-PET SUVR and Tau-PET Braak stages, suggesting the two dietary habits may be helpful for postponing the progression of AD. Correspondingly, the follow-up study exhibited the above habits prevented the decline of MoCA-BC, which supports the above speculation. The mechanisms may be related to the nutrients rich in vegetables and fruits. For example, flavonoids, which are rich in dark vegetables and berries, have the ability to reduce Aβ and Tau burden in AD models [44,45]. In addition, it has been shown that folate deficiency induces the accumulation of Aβ and phosphorylated Tau protein in the brain and sufficient intake of folate may protect against AD [46,47]. Actually, high consumption of dark vegetables like spinach and carrot can help supplement of folate. Furthermore, the phenolic compounds like ellagic acid and pelargonidin primarily found in berries can reduce Aβ aggregation [48]. In addition, both grape skin anthocyanin extract and grape seed proanthocyanidin extract are capable of improving the cognitive function and reducing Aβ and Tau pathology [49].

Compared with the CSF biomarkers, blood-based biomarkers have the advantages of non-invasive and more accessible, which develop rapidly and are promising for future real-world clinical implementation to detect the pathophysiology of AD [50]. Notably, among the plasma biomarkers tested in our study, Aβ42/40 and p-Tau181 have significance especially in CU adults. It has been demonstrated that plasma Aβ42/40 may be considered as a potential predictor for the conversion to clinical MCI and dementia due to AD in CU subjects [51]. Meanwhile, high plasma p-Tau181 is revealed to carry an increased risk of AD dementia in CU individuals with Aβ positive compared with those with Aβ negative [52]. We demonstrated the associations between the consumption of vegetables and fruits and the above biomarkers. Considering that the biomarkers correlate with AD pathology and play roles in predicting cognitive decline in CU population, it adds the evidence that these habits may be more essential to CU individuals. As blood-based biomarkers are capable of accelerating and scaling up the diagnostic pathways of early symptomatic AD in at risk CU individuals [53], we may monitor the effects of dietary invention by using these biomarkers. As dietary habit is a kind of modifiable lifestyle which could be a tractable target for early intervention to reduce AD pathogenesis, we speculate that early adjustment to these beneficial dietary habits demonstrated by the current work may be helpful for delaying the pathological process of AD.

Several limitations of this study should be considered when interpreting the results. It was a single-center study with a relatively small sample size and shorter follow-up time. Meanwhile, not all CU participants with positive Aβ-PET underwent the tests of plasma biomarkers and Tau-PET and follow-up study. In addition, the proportion of participants with amyloid-PET positive was higher than the general population since the majority of participants were from the specialist cognitive clinic. It may be explained from two aspects. On one hand, CI participants who already suffered from clinical symptoms, especially those with the development similar to AD, had stronger willingness to complete amyloid-PET examination in order to acquire more precise diagnosis and treatment. Accordingly, the positivity of Aβ-PET was more common in such population. On the other hand, in CU participants who had completed the questionnaires, those also willing to undergo amyloid-PET examination were often diagnosed subjective cognitive decline with abnormal plasma biomarkers and/or carrying *APOE* ε4 alleles. As a result, Aβ-PET exhibited higher positive rate also in such CU participants. Nevertheless, the high positive rate of Aβ-PET may cause selection bias and influence the generalizability of our study. Moreover, the self-reported questionnaire only included the consumption of fresh vegetables and fruits. The intake of other forms such as canned, frozen and dried vegetables and fruits, juice and wine as well as other dietary habits were not investigated. Besides, the biomarkers were plasma analytes instead of being enriched for neuronal origin using an exosome platform and the CSF biomarkers as gold standard had not been tested. Further prospective study with long-term follow-up in a consecutive community population containing the improvement of the above limitations should be conducted to validate and generalize our findings.

In conclusion, our study including 1433 participants demonstrates that most of the intake habits of fresh vegetables and fruits are different between CU and CI participants of Chinese population. Furthermore, higher consumption of vegetables, dark vegetables, fruits, berries and grapes is associated with PET and plasma biomarkers in the CU participants with Aβ-PET (+). Especially, higher intakes of fruits (>100 g/d) and grapes (>100 g/d) correlate with both lower Aβ-PET SUVR and Tau-PET Braak stages as well as inversely with cognitive decline after a two-year follow-up. The beneficial vegetable and fruit consumption may be different in the subgroups stratified by *APOE* ε4 genotype. Adjusting to these dietary habits may help reduce the risk of AD development.

## Funding

This work was supported by the National Natural Science Foundation of China (82171198; 82071472; 81901102) and Shanghai Municipal Science and Technology Major Project (No. 2018SHZDZX01).

## Data availability

The datasets collected and/or analyzed during this study are available from the corresponding author on reasonable request.

## Ethical standards

This study was approved by the ethical standards of the Ethics Committee of Shanghai Sixth People's Hospital Affiliated to Shanghai Jiao Tong University School of Medicine (No. 2019–032), and written informed consent was provided by each patient's next of kin/participant.

## CRediT authorship contribution statement

**Heling Chu:** Writing – review & editing, Writing – original draft, Methodology, Investigation, Funding acquisition, Formal analysis. **Chuyi Huang:** Writing – review & editing, Writing – original draft, Investigation, Funding acquisition. **Fang Xie:** Writing – review & editing, Validation, Software, Methodology. **Qihao Guo:** Writing – review & editing, Visualization, Validation, Supervision, Funding acquisition, Conceptualization.

## Declaration of competing interest

The authors declare that they have no known competing financial interests or personal relationships that could have appeared to influence the work reported in this paper.

## Appendix Supplementary materials

Image, application 1
