# Supplementary material for: The associations between fresh vegetable and fruit consumption and plasma and PET biomarkers in preclinical Alzheimer's disease: A cross-sectional and longitudinal study of Chinese population
Source: J Prev Alzheimers Dis. 2025 Jan 30;12(5):100076. doi: 10.1016/j.tjpad.2025.100076 (PMC12184019; doi:10.1016/j.tjpad.2025.100076)
Supplement: Supplementary file 1 [file mmc1.docx]

**eTable 1.** The questionnaire of vegetable and fruit consumption.

| 1. How many fresh vegetables do you consume every day? 2. >300 g 3. 200-300 g 4. 100-200 g 5. <100 g |
| --- |
| 1. How often do you consume dark vegetables (e.g. spinach, water spinach, broccoli, chrysanthemum coronarium, carrot, pumpkin, chili, tomato, purple cabbage, amaranth)? 2. ≥4 day/week 3. 2-3 day/week 4. 1 day/week 5. <1 day/week |
| 1. How many dark vegetables do you consume every day? 2. >150 g 3. 100-150 g 4. 50-100 g 5. <50 g |
| 1. How many fresh fruits do you consume every day? 2. >200 g 3. 100-200 g 4. <100 g 5. No |
| 1. How many berries (e.g. grape, strawberry, kiwifruit, pomegranate, blueberry, mulberry, cranberry, carambola, guava) do you consume every day during the season? 2. >200 g 3. 100-200 g 4. <100 g |
| 1. How many grapes do you consume every day during the season? 2. >200 g 3. 100-200 g 4. <100 g |

**eTable 2.** Demographic and clinical characteristics of all included participants.

| Characteristics | Aβ-PET | | | | | | No Aβ-PET | | |
| --- | --- | --- | --- | --- | --- | --- | --- | --- | --- |
|  | Positive | | | Negative | | |  |  |  |
|  | CU  (n=177) | CI  (n=168) | *P* | CU  (n=206) | CI  (n=147) | *P* | CU  (n=325) | CI  (n=410) | *P* |
| Age, y, mean (SD) | 65.3 (7.1) | 68.0 (7.6) | 0.001 | 64.5 (8.2) | 66.1 (6.9) | 0.056 | 64.7 (9.1) | 70.1 (7.0) | <0.001 |
| Sex, male, n (%) | 78 (44.1) | 69 (41.1) | 0.574 | 66 (32.0) | 56 (38.1) | 0.238 | 92 (28.3) | 132 (32.2) | 0.246 |
| Education, y, median (IQR) | 12 (10-15) | 11 (8-13) | <0.001 | 12 (10-14) | 10 (8-13) | <0.001 | 13 (11-15) | 10 (9-12) | <0.001 |
| BMI, kg/m^2^, mean (SD) | 23.0 (2.7) | 23.0 (3.1) | 0.870 | 23.8 (3.2) | 23.8 (3.1) | 0.884 | 23.4 (3.5) | 23.7 (3.3) | 0.277 |
| Smoking, n (%) | 27 (15.3) | 24 (14.3) | 0.551 | 23 (11.2) | 26 (17.7) | 0.076 | 33 (10.2) | 50 (12.2) | 0.290 |
| Alcohol consumption, n (%) | 33 (18.6) | 31 (18.5) | 0.587 | 41 (19.9) | 27 (18.4) | 0.715 | 42 (12.9) | 63 (15.4) | 0.332 |
| Hypertension, n (%) | 56 (31.6) | 59 (35.1) | 0.493 | 67 (32.5) | 50 (34.0) | 0.769 | 121 (37.2) | 151 (36.8) | 0.911 |
| Diabetes mellitus, n (%) | 22 (12.4) | 18 (10.7) | 0.619 | 24 (11.7) | 17 (11.6) | 0.980 | 32 (9.8) | 54 (13.2) | 0.164 |
| Hyperlipidemia, n (%) | 21 (11.9) | 26 (15.5) | 0.328 | 42 (20.4) | 31 (21.1) | 0.873 | 56 (17.2) | 70 (17.1) | 0.955 |
| Coronary heart disease, n (%) | 13 (7.3) | 11 (6.5) | 0.771 | 22 (10.7) | 14 (9.5) | 0.724 | 17 (5.2) | 33 (8.0) | 0.132 |
| MMSE, median (IQR) | 28 (27-29) | 23 (17-26) | <0.001 | 28 (27-29) | 26 (23-27) | <0.001 | 28 (27-29) | 24 (19-27) | <0.001 |
| MoCA-BC, median (IQR) | 25 (23-27) | 16 (13-21) | <0.001 | 26 (24-27) | 21 (16-23) | <0.001 | 26 (24-28) | 18 (13-22) | <0.001 |
| Vegetable and fruit consumption |  |  |  |  |  |  |  |  |  |
| Vegetable consumption, median (IQR) | 2 (2-3) | 3 (2-3) | <0.001 | 2 (2-3) | 3 (2-3) | 0.185 | 2 (2-3) | 2 (2-3) | 0.116 |
| Vegetable consumption>200 g/d, n (%) | 105 (59.3) | 67 (39.9) | <0.001 | 118 (57.3) | 72 (49.0) | 0.123 | 186 (57.2) | 217 (52.9) | 0.259 |
| Frequency of Dark vegetable consumption, median (IQR) | 1 (1-2) | 1 (1-2) | 0.907 | 1 (1-2) | 1 (1-2) | 0.603 | 1 (1-2) | 1 (1-2) | 0.176 |
| Dark vegetable consumption≥ 2 d/week, n (%) | 167 (94.4) | 155 (92.3) | 0.437 | 181 (87.9) | 123 (83.7) | 0.262 | 299 (92.0) | 349 (85.1) | 0.005 |
| Dark vegetable consumption, median (IQR) | 1 (1-2) | 2 (1-2) | 0.049 | 1 (1-2) | 1 (1-2) | 0.787 | 2 (1-2) | 2 (1-2) | 0.942 |
| Dark vegetable consumption>100 g/d, n (%) | 150 (84.7) | 137 (81.5) | 0.427 | 165 (80.1) | 116 (78.9) | 0.785 | 251 (77.2) | 308 (75.1) | 0.543 |
| Fruit consumption, median (IQR) | 2 (2-3) | 2 (2-3) | 0.001 | 2 (2-3) | 2 (2-3) | 0.012 | 2 (2-3) | 2 (2-3) | 0.075 |
| Fruit consumption>100 g/d, n (%) | 120 (67.8) | 85 (50.6) | 0.001 | 151 (73.3) | 86 (58.5) | 0.004 | 222 (68.3) | 255 (62.2) | 0.093 |
| Berry consumption, median (IQR) | 3 (2-3) | 3 (2-3) | <0.001 | 3 (2-3) | 3 (2-3) | 0.034 | 2 (2-3) | 3 (2-3) | <0.001 |
| Berry consumption>100 g/d, n (%) | 88 (49.7) | 50 (29.8) | <0.001 | 99 (48.1) | 53 (36.1) | 0.025 | 190 (58.5) | 166 (40.5) | <0.001 |
| Grape consumption, median (IQR) | 3 (2-3) | 3 (2-3) | <0.001 | 3 (2-3) | 3 (2-3) | 0.017 | 3 (2-3) | 3 (2-3) | 0.011 |
| Grape consumption>100 g/d, n (%) | 84 (47.5) | 47 (28.0) | <0.001 | 84 (40.8) | 41 (27.9) | 0.013 | 156 (48.0) | 159 (38.8) | 0.013 |

BMI indicates body mass index; CU, cognitively unimpaired; CI, cognitively impaired; IQR, interquartile range; MMSE, Mini-Mental State Examination; MoCA-BC, Chinese version of Montreal Cognitive Assessment-Basic; SD, standard deviation

**eTable 3.** Spearman’s correlation and multiple linear regression analyzes of the association between vegetable and fruit consumption and plasma biomarkers in cognitively unimpaired participants with Aβ-PET (+).

| Variables | Biomarkers | Spearman’s correlation | Multiple linear regression | | | |
| --- | --- | --- | --- | --- | --- | --- |
|  |  | Correlation coefficient  (*P* value) | β | 95% CI | SE | *P* value |
| Vegetable consumption | Aβ42 | -0.059 (0.471) | / | / | / | / |
|  | Aβ40 | -0.026 (0.750) | / | / | / | / |
|  | Aβ42/40 | -0.056 (0.499) | / | / | / | / |
|  | t-Tau | -0.002 (0.978) | / | / | / | / |
|  | p-Tau-181 | -0.105 (0.205) | / | / | / | / |
|  | NfL | -0.207 (0.015) | / | / | / | / |
| Vegetable consumption>200 g/d | Aβ42 | -0.097 (0.239) | / | / | / | / |
|  | Aβ40 | -0.054 (0.514) | / | / | / | / |
|  | Aβ42/40 | -0.029 (0.727) | / | / | / | / |
|  | t-Tau | -0.042 (0.613) | / | / | / | / |
|  | p-Tau-181 | -0.079 (0.341) | / | / | / | / |
|  | NfL | -0.171 (0.045) | / | / | / | / |
| Frequency of Dark vegetable consumption | Aβ42 | 0.003 (0.976) | / | / | / | / |
|  | Aβ40 | -0.060 (0.464) | / | / | / | / |
|  | Aβ42/40 | 0.027 (0.739) | / | / | / | / |
|  | t-Tau | 0.010 (0.902) | / | / | / | / |
|  | p-Tau-181 | -0.102 (0.222) | / | / | / | / |
|  | NfL | -0.167 (0.050) | / | / | / | / |
| Dark vegetable consumption≥ 2 d/week | Aβ42 | -0.031 (0.708) | / | / | / | / |
|  | Aβ40 | -0.151 (0.066) | / | / | / | / |
|  | Aβ42/40 | 0.077 (0.346) | / | / | / | / |
|  | t-Tau | -0.090 (0.285) | / | / | / | / |
|  | p-Tau-181 | -0.198 (0.016) | / | / | / | / |
|  | NfL | -0.134 (0.117) | / | / | / | / |
| Dark vegetable consumption | Aβ42 | -0.182 (0.025) | / | / | / | / |
|  | Aβ40 | -0.163 (0.046) | -21.349 | -35.959 to -6.739 | 7.378 | 0.005 |
|  | Aβ42/40 | -0.007 (0.934) | / | / | / | / |
|  | t-Tau | -0.084 (0.318) | / | / | / | / |
|  | p-Tau-181 | -0.199 (0.016) | -0.331 | -0.560 to -0.101 | 0.258 | 0.005 |
|  | NfL | -0.125 (0.143) | / | / | / | / |
| Dark vegetable consumption>100 g/d | Aβ42 | -0.065 (0.432) | / | / | / | / |
|  | Aβ40 | -0.073 (0.375) | / | / | / | / |
|  | Aβ42/40 | -0.004 (0.960) | / | / | / | / |
|  | t-Tau | -0.160 (0.055) | / | / | / | / |
|  | p-Tau-181 | -0.244 (0.003) | -0.719 | -1.249 to -0.189 | 0.268 | 0.008 |
|  | NfL | -0.135 (0.114) | / | / | / | / |
| Fruit consumption | Aβ42 | -0.023 (0.781) | / | / | / | / |
|  | Aβ40 | -0.054 (0.514) | / | / | / | / |
|  | Aβ42/40 | 0.088 (0.285) | / | / | / | / |
|  | t-Tau | -0.181 (0.030) | -0.260 | -0.495 to -0.025 | 0.119 | 0.031 |
|  | p-Tau-181 | -0.191 (0.021) | / | / | / | / |
|  | NfL | -0.357 (<0.001) | -1.845 | -3.447 to -0.242 | 0.809 | 0.024 |
| Fruit consumption>100 g/d | Aβ42 | -0.081 (0.323) | / | / | / | / |
|  | Aβ40 | -0.063 (0.446) | / | / | / | / |
|  | Aβ42/40 | 0.037 (0.657) | / | / | / | / |
|  | t-Tau | -0.146 (0.080) | / | / | / | / |
|  | p-Tau-181 | -0.147 (0.076) | / | / | / | / |
|  | NfL | -0.232 (0.006) | / | / | / | / |
| Berry consumption | Aβ42 | -0.120 (0.143) | / | / | / | / |
|  | Aβ40 | -0.156 (0.056) | / | / | / | / |
|  | Aβ42/40 | 0.058 (0.478) | / | / | / | / |
|  | t-Tau | -0.139 (0.096) | / | / | / | / |
|  | p-Tau-181 | -0.217 (0.009) | -0.393 | -0.635 to -0.151 | 0.122 | 0.002 |
|  | NfL | -0.345 (<0.001) | / | / | / | / |
| Berry consumption>100 g/d | Aβ42 | -0.110 (0.180) | / | / | / | / |
|  | Aβ40 | -0.163 (0.046) | / | / | / | / |
|  | Aβ42/40 | 0.075 (0.363) | / | / | / | / |
|  | t-Tau | -0.109 (0.192) | / | / | / | / |
|  | p-Tau-181 | -0.149 (0.074) | / | / | / | / |
|  | NfL | -0.272 (0.001) | / | / | / | / |
| Grape consumption | Aβ42 | -0.069 (0.400) | / | / | / | / |
|  | Aβ40 | -0.414 (0.086) | / | / | / | / |
|  | Aβ42/40 | 0.094 (0.253) | / | / | / | / |
|  | t-Tau | -0.081 (0.333) | / | / | / | / |
|  | p-Tau-181 | -0.258 (0.002) | -0.361 | -0.617 to -0.104 | 0.129 | 0.006 |
|  | NfL | -0.264 (0.002) | / | / | / | / |
| Grape consumption>100 g/d | Aβ42 | -0.068 (0.409) | / | / | / | / |
|  | Aβ40 | -0.138 (0.092) | / | / | / | / |
|  | Aβ42/40 | 0.101 (0.221) | / | / | / | / |
|  | t-Tau | -0.072 (0.393) | / | / | / | / |
|  | p-Tau-181 | -0.213 (0.010) | -0.545 | -0.928 to -0.162 | 0.193 | 0.006 |
|  | NfL | -0.209 (0.014) | / | / | / | / |

Aβ indicates amyloid-β; CI, confidence intervals; NfL, neurofilament light chain; PET, positron emission tomography; SE, standardized estimate

**eTable 4.** Spearman’s correlation and multiple linear regression analyzes of the associations between vegetable and fruit consumption and the biomarkers in the cross-sectional longitudinal study of the cognitively unimpaired *APOE* ε4 carriers or

noncarriers with Aβ-PET (+) ^*^.

| Variables | Biomarkers | Spearman’s correlation | Multiple linear regression | | | |
| --- | --- | --- | --- | --- | --- | --- |
|  |  | Correlation coefficient  (*P* value) | β | 95% CI | SE | *P* value |
| *APOE* ε4 carrier | | | | | | |
| Dark vegetable consumption | Aβ42/40 | 0.418 (0.011) | 0.009 | 0.004 to 0.013 | 0.002 | <0.001 |
| Fruit consumption | Tau-PET Braak stages | -0.595 (0.006) | -1.167 | -1.833 to -0.501 | 0.303 | 0.003 |
| Fruit consumption>100 g/d | Tau-PET Braak stages | -0.562 (0.010) | -1.800 | -3.043 to -0.557 | 0.565 | 0.009 |
| Berry consumption | Aβ-PET SUVR | -0.437 (0.003) | -0.176 | -0.278 to -0.073 | 0.050 | 0.001 |
| Berry consumption>100 g/d | Aβ-PET SUVR | -0.406 (0.006) | -0.250 | -0.402 to -0.098 | 0.075 | 0.002 |
| Grape consumption | Aβ-PET SUVR | -0.347 (0.019) | -0.136 | -0.258 to -0.015 | 0.060 | 0.015 |
|  | Tau-PET Braak stages | -0.518 (0.019) | -0.989 | -1.903 to -0.076 | 0.415 | 0.036 |
| Follow-up | | | | | | |
| Berry consumption | dMoCA | 0.787 (0.001) | 5.458 | 0.019 to 10.898 | 2.405 | 0.049 |
|  | CI% | 0.645 (0.013) | 0.692 | 0.316 to 1.069 | 0.163 | 0.003 |
| Berry consumption>100 g/d | dMoCA | 0.787 (0.001) | 5.458 | 0.019 to 10.898 | 2.405 | 0.049 |
|  | CI% | 0.645 (0.013) | 0.692 | 0.316 to 1.069 | 0.163 | 0.003 |
| *APOE* ε4 non-carrier | | | | | | |
| Frequency of Dark vegetable consumption | NfL | -0.205 (0.037) | -2.236 | -4.506 to -0.147 | 1.094 | 0.037 |
|  | Aβ-PET SUVR | -0.277 (0.003) | -0.070 | -0.129 to -0.011 | 0.030 | 0.021 |
| Dark vegetable consumption≥ 2 d/week | NfL | -0.194 (0.049) | -7.111 | -12.572 to -1.651 | 2.742 | 0.011 |
|  | Aβ-PET SUVR | -0.186 (0.046) | -0.195 | -0.341 to -0.050 | 0.073 | 0.009 |
| Dark vegetable consumption | Aβ42 | -0.208 (0.026) | -0.977 | -1.910 to -0.045 | 0.469 | 0.040 |
|  | p-Tau-181 | -0.196 (0.039) | -0.383 | -0.646 to -0.120 | 0.132 | 0.005 |
|  | Aβ-PET SUVR | -0.199 (0.033) | -0.061 | -0.107 to -0.015 | 0.023 | 0.009 |
| Dark vegetable consumption >100 g/d | p-Tau-181 | -0.244 (0.003) | -0.919 | -1.511 to -0.327 | 0.298 | 0.003 |
| Fruit consumption | NfL | -0.403 (<0.001) | -2.190 | -3.730 to -0.649 | 0.774 | 0.006 |
|  | Aβ-PET SUVR | -0.374 (<0.001) | -0.052 | -0.095 to -0.010 | 0.021 | 0.016 |
| Berry consumption | NfL | -0.412 (<0.001) | -2.168 | -4.026 to -0.310 | 0.933 | 0.023 |
|  | p-Tau-181 | -0.233 (0.013) | -0.434 | -0.717 to -0.151 | 0.142 | 0.003 |
|  | Aβ-PET SUVR | -0.430 (<0.001) | -0.103 | -0.152 to -0.053 | 0.025 | <0.001 |
| Berry consumption>100 g/d | Aβ-PET SUVR | -0.392 (<0.001) | -0.126 | -0.203 to -0.048 | 0.039 | 0.002 |
| Grape consumption | p-Tau-181 | -0.283 (0.003) | -0.361 | -0.654 to -0.068 | 0.147 | 0.016 |
|  | Aβ-PET SUVR | -0.351 (<0.001) | -0.092 | -0.144 to -0.041 | 0.026 | 0.001 |
| Grape consumption>100 g/d | p-Tau-181 | -0.242 (0.010) | -0.596 | -1.042 to -0.150 | 0.224 | 0.009 |
|  | Aβ-PET SUVR | -0.340 (<0.001) | -0.124 | -0.202 to -0.047 | 0.039 | 0.002 |
| Follow-up | | | | | | |
| Vegetable consumption | dSUVR | -0.589 (0.006) | -0.146 | -0.202 to -0.091 | 0.025 | <0.001 |
| Vegetable consumption>200 g/d | dSUVR | -0.731 (<0.001) | -0.356 | -0.487 to -0.225 | 0.060 | <0.001 |
| Frequency of Dark vegetable consumption | dMoCA | 0.426 (0.004) | 2.072 | 1.046 to 3.097 | 0.501 | <0.001 |
| Dark vegetable consumption≥ 2 d/week | dMoCA | 0.382 (0.010) | 3.846 | 1.144 to 6.548 | 1.319 | 0.007 |
| Dark vegetable consumption | dMoCA | 0.401 (0.006) | 1.332 | 0.460 to 2.203 | 0.426 | 0.004 |
|  | CI% | -0.343 (0.021) | -0.134 | -0.232 to -0.037 | 0.048 | 0.009 |
| Dark vegetable consumption>100 g/d | dMoCA | 0.335 (0.024) | 2.500 | 0.063 to 4.937 | 1.190 | 0.045 |
|  | CI% | -0.354 (0.017) | -0.311 | -0.590 to -0.033 | 0.136 | 0.030 |
| Fruit consumption>100 g/d | dMoCA | 0.321 (0.032) | 2.150 | 0.087 to 4.213 | 1.007 | 0.042 |
| Berry consumption>100 g/d | dSUVR | -0.723 (<0.001) | -0.192 | -0.360 to -0.025 | 0.077 | 0.027 |

* Only the associations of *P* values<0.05 in multiple linear regression analyzes were listed.

Aβ indicates amyloid-β; CI, confidence intervals; CI%, conversion rate of cognitive impairment; “d”, difference between the follow-up and baseline data; MoCA-BC, Chinese version of Montreal Cognitive Assessment-Basic; NfL, neurofilament light chain; PET, positron emission tomography; SE, standardized estimate; SUVR, standard uptake value ratio


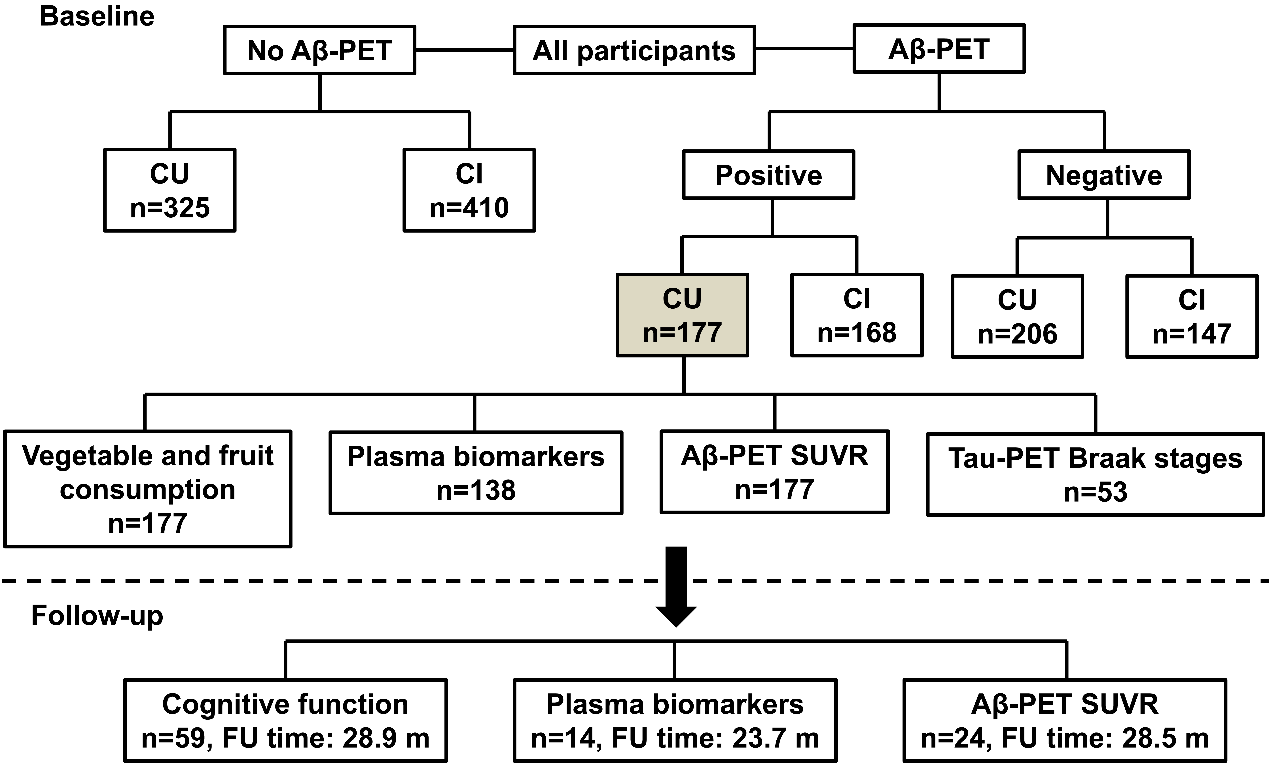


**eFigure 1.** The groups and study protocol. Aβ indicates amyloid-β; CI, cognitive impairment; CU, cognitive unimpaired; FU, follow-up; PET, positron emission tomography; SUVR, standard uptake value ratio
